# Loss of mural cell-derived laminin aggravates hemorrhagic brain injury

**DOI:** 10.1186/s12974-020-01788-3

**Published:** 2020-04-06

**Authors:** Jyoti Gautam, Lingling Xu, Abhijit Nirwane, Benjamin Nguyen, Yao Yao

**Affiliations:** Department of Pharmaceutical and Biomedical Sciences, University of Georgia, 240 W Green Street, Athens, GA 30602 USA

**Keywords:** Intracerebral hemorrhage, Blood-brain barrier, Mural cells, Edema, Laminin

## Abstract

**Background:**

Mural cells synthesize and deposit laminin to the basement membrane. To investigate the function of mural cell-derived laminin, we generated a mutant mouse line lacking mural cell-derived laminin (termed PKO). In a previous study, we showed that the PKO mice were grossly normal under homeostatic condition, but developed blood-brain barrier (BBB) breakdown with advanced age (> 8 months), suggesting that these mutants are intrinsically weak. Based on these findings, we hypothesized that PKO mice have exacerbated injuries in pathological conditions.

**Methods:**

Using collagenase-induced intracerebral hemorrhage (ICH) as an injury model, we examined various stroke outcomes, including hematoma volume, neurological function, neuronal death, BBB integrity, paracellular/transcellular transport, inflammatory cell infiltration, and brain water content, in PKO mice and their wildtype littermates at young age (6–8 weeks). In addition, transmission electron microscopy (TEM) analysis and an in vitro ICH model were used to investigate the underlying molecular mechanisms.

**Results:**

Compared to age-matched wildtype littermates, PKO mice display aggravated stroke outcomes, including larger hematoma size, worse neurological function, increased neuronal cell death, enhanced BBB permeability, increased transcytosis, and elevated inflammatory cell infiltration. These mutants also exhibit high baseline brain water content independent of aquaporin-4 (AQP4). In addition, mural cell-derived laminin significantly reduced caveolin-1 without affecting tight junction proteins in the in vitro ICH model.

**Conclusions:**

These results suggest that mural cell-derived laminin attenuates BBB damage in ICH via decreasing caveolin-1 and thus transcytosis, regulates brain water homeostasis, and plays a beneficial role in ICH.

## Background

Stroke is a leading cause of death and long-term disability in the USA [1, 2]. Although ICH accounts for only 10–15% of all strokes, it has extremely high mortality and disability rates and there is no effective treatment for this devastating disease so far [1, 3]. Emerging evidence suggests that the neurovascular unit plays an important role in stroke pathogenesis [4, 5]. The neurovascular unit consists of many different cell types and a non-cellular component—the basement membrane (BM) [6–8]. By interacting with different cell types via integrins and other receptors, the BM contributes to the structural and functional integrity of the neurovascular unit [9, 10]. It has been reported that integrin expression is substantially altered after stroke and integrins play an important role in stroke pathogenesis [9, 11–13]. Compared to integrins, the BM is relatively understudied. Although BM dissolution is observed after stroke, how each BM component changes in stroke is controversial [6, 9, 14]. In addition, the function of BM in the pathogenesis of stroke remains largely unknown.

Laminin, a trimeric protein composed of α, β, and γ subunits is the only constituent required for BM assembly [9, 15, 16]. So far, five α, four β, and three γ genetic variants have been identified [9, 15, 16]. Different combinations of these subunits generate a large number of laminin isoforms. However, only 20 isoforms have been identified or proposed based on experimental data [9, 15, 17]. At the neurovascular unit, laminin is mainly synthesized by astrocytes, brain microvascular endothelial cells (BMECs), and pericytes [9]. Interestingly, these cells make different laminin isoforms. For example, astrocytes generate laminin-α2β1γ1 (-211) [9, 18–20], BMECs mainly make laminin-α4β1γ1 (-411) and -α5β1γ1 (-511) [9, 21–23], while pericytes predominantly synthesize α4/α5- and γ1-containing laminins [9, 24]. Located at the interface of the circulation system and the CNS [9, 15], laminin is well positioned to regulate BBB permeability.

Using conditional knockout mice and adenovirus expressing Cre under GFAP promoter, we have shown that loss of astrocytic laminin leads to BBB breakdown and age-dependent ICH [20, 25]. Echoed with this finding, laminin-α2^−/−^ mice show BBB disruption [19]. These results suggest an indispensable role of astrocytic laminin (laminin-211) in BBB maintenance under homeostatic condition. Using endothelium-specific laminin-α5 knockout mice, we and others have demonstrated that endothelial laminin-α5 is dispensable for BBB maintenance under homeostatic condition but promotes BBB recovery after ICH [26, 27].

To investigate the function of pericyte-derived laminin in BBB maintenance, we generated a mural cell-specific conditional knockout mouse line (laminin-γ1^flox/flox^:PDGFRβ-Cre^+^, termed PKO) by crossing the laminin-γ1 floxed mice with the PDGFRβ-Cre line. Laminin-γ1 was chosen for the following two reasons: (1) among all three γ subunits, laminin-γ1 is highly expressed in pericytes [9, 24, 28] and (2) pericytes mainly express α4/α5- and γ1-containing laminins, and ablating laminin-γ1 leads to loss of both laminin-α4 and laminin-α5 in pericytes [28]. Under C57Bl6-FVB mixed background, these PKO mutants display hydrocephalus and BBB breakdown with incomplete penetrance [24]. To determine whether the BBB disruption phenotype is due to loss of mural cell-derived laminin or secondary to hydrocephalus, which can compromise BBB integrity, we crossed the PKO mice into C57Bl6 dominant background, in which they fail to develop hydrocephalus [28]. Under C57Bl6 dominant background, the PKO mice have intact BBB integrity at young age (< 4 months) and develop mild BBB compromise at old age (8–15 months) [28], possibly due to diminished compensation by other laminin isoforms at old age. These results suggest that mural cell-derived laminin also contributes to BBB maintenance but to a lesser extent.

We reason that, in conditions with compromised laminin compensation (e.g., increased laminin degradation), the PKO mice will show exacerbated outcomes even at young age. Here, we tested this hypothesis using ICH as a model, where laminin levels are significantly reduced [6].

## Materials and methods

### Animals

Laminin-γ1^flox/flox^ mice were crossed with the PDGFRβ-Cre^+^ line to generate PKO (laminin-γ1^flox/flox^:Pdgfrβ-Cre^+^) mice. Laminin-γ1^flox/flox^ mice and heterozygotes (laminin-γ1^flox/+^:Pdgfrβ-Cre^+^) were used as littermate controls for PKO mice. All mice were maintained in C57Bl6 dominant background and mice of both genders at 6–8 weeks old were used in this study. The validation and characterization of the PKO mice were reported in previous studies [24, 29]. All mice were maintained in the animal facility at the University of Georgia with free access to water and food. Experimental procedures were conducted in accordance with the NIH guide for care and use of animals and were approved by the Institutional Animal Care and Use Committee (IACUC) at the University of Georgia. Experiments were conducted and reported per the ARRIVE guidelines.

### ICH model

Control and PKO mice were anesthetized by intraperitoneal injection of avertin (500 mg/kg of body weight) and placed on the stereotaxic frame (Stoelting Co., IL, USA). Through a hole drilled in the skull, collagenase (type VII-S; Sigma, St. Louis, USA; 0.15 U in 0.7 μl saline) was injected into the striatum (0.2 mm posterior to bregma, 2.4 mm lateral from the midline, and 3.7 mm in depth) using a 1-μl syringe (Hamilton) controlled by a stereotaxic instrument over 5 min. After injection, the needle was kept in place for 10 min to prevent reflux. Mice injected with equal amount of saline were used as sham controls. At various time points after injury, mice were transcardially perfused with either PBS or PBS followed by 4% paraformaldehyde (PFA) [30–35]. For the former, the brains were frozen in dry ice, cryosectioned, and stored at − 80 °C. For the latter, the brains were post-fixed in 4% PFA overnight at 4 °C, immersed in 30% sucrose solution for 24 h, cryosectioned, and stored at − 80 °C.

### Neurological deficit

Mice were scored for neurological deficits at various time points after injury using a modified scoring system [30, 31, 36]. In this system, six properties (body symmetry, gait, climbing, circling behavior, front limb symmetry, and compulsory circling) were graded from 0 to 4, establishing a maximum score of 24. Higher scores indicate more severe neurological deficits. Investigators performing this test were blinded to the genotype of mice.

### Histology

Hematoxylin staining was performed as described previously [30–32]. Briefly, sections were washed twice in PBS and kept in hematoxylin for 15 min at room temperature. After two washes in tap water, the sections were incubated in 70% ethanol with 1% hydrochloric acid for 3 s, followed by three washes in tap water. Then, the sections were dehydrated in graded alcohol (70%, 80%, 90%, 100%, and 100%), cleared in xylene, and mounted with DPX. Images were taken under a Nikon Eclipse Ti microscope. Hematoma size was quantified using serial coronal sections as described previously [30, 36]. Briefly, 20-μm-thick serial coronal sections were cut using a cryostat (Microm HM 550, Thermo Scientific, USA). Eight sections evenly distributed along the rostral-to-caudal axis were collected from each brain. Hematoma areas (mm^2^) from serial coronal sections were added, and the injury volume (mm^3^) was calculated as follows: injury volume = measured area × section thickness.

Fluoro-Jade C (FJC) staining was performed using a standard protocol as described previously [30, 37]. Briefly, sections were incubated in 1% NaOH in 80% ethanol for 5 min, washed in 70% ethanol and distilled water, and incubated in 0.06% KMnO_4_ for 10 min. After extensive washes in water, the sections were transferred to 0.0001% FJC (in 0.1% acetic acid) for 10 min. Then, the sections were rinsed in water, dried at 56 °C, cleared in xylene, and mounted with DPX. Images were taken from peri-hematoma regions using a Nikon Eclipse Ti microscope. FJC^+^ cells were counted in six to eight sections (three fields/section) immediately adjacent to the hematoma as described previously [30–32]. At least three animals/group/time point were used for quantification.

### Immunohistochemistry

Brain sections were fixed in 4% PFA for 15 min. After extensive washes with PBS, the sections were incubated in blocking buffer (1 % BSA in PBS containing 0.3% normal donkey serum and 0.3 % Triton X-100) for 1 h at room temperature. Next, the sections were incubated with primary antibodies [rat-anti-CD31 (1:200, BD Biosciences, 553370), mouse anti-claudin-5 (1:200, Invitrogen, USA, 35-2500), rabbit anti-ZO-1 (1:400, Thermofisher, USA, 61-7300), rabbit anti-caveolin-1 (1:500, Cell Signaling, 3238S), rabbit anti-PDGFRβ (1:200, Cell Signaling, 3169), rabbit anti-AQP4 (1:500, Millipore, USA, AB3594), rat anti-Ly6G (1:200; Biolegend, USA, 108402), rat anti-CD3 (1:200, eBioscience, USA, 14–0032-82), rat anti-CD11b (1:200, BD Biosciences, 553309), mouse anti-glial fibrillary acidic protein (GFAP, 1:200, BD Bioscience, USA, 556327), and rabbit anti-Iba1 (1:500; Wako Inc, USA, 019-19741)] overnight at 4 °C. After extensive washes, the sections were incubated with appropriate fluorescent secondary antibodies. After extensive washes, the sections were mounted with fluoromount-G with DAPI. Images were taken from peri-hematoma regions using a Nikon Eclipse Ti microscope or LSM710 confocal microscope.

### Image analyses

For claudin-5/ZO-1 coverage, claudin-5-, ZO-1-, and CD31-positive fluorescent areas were determined using the ImageJ area measurement tool. Claudin-5/ZO-1 coverage was determined as the percentage (%) of claudin-5/ZO-1-positive fluorescent area covering CD31-positive capillary area. Average claudin5/ZO-1 length was determined by measuring the length of claudin5^+^ and ZO-1^+^ vessels using the NIH ImageJ “Neuro J” plug-in length analysis tool. For pericyte coverage, PDGFRβ- and CD31-positive fluorescent areas were determined using the ImageJ area measurement tool. Pericyte coverage was determined as the percentage (%) of PDGFRβ-positive fluorescent area covering CD31-positive capillary area, as previously described [38]. For AQP4 coverage, AQP4- and CD31-positive fluorescent areas were determined using the ImageJ area measurement tool. AQP4 coverage was determined as the percentage (%) of AQP4-positive fluorescent area covering CD31-positive capillary area. For caveolin-1 expression, mean fluorescence intensity was used. For inflammatory cell infiltration, total numbers of Ly6G^+^/CD3^+^/CD11b^+^ cells were counted. For microglial number, Iba1^+^ cells were counted. For astrocyte activation, GFAP mean fluorescence intensity was used. For quantifications, three random fields from each section, 8 serial sections along the rostral-to-caudal axis for each brain, and 3–5 animals were used. All data analyses were performed on z-projection (15–20 μm) images by a blinded investigator.

### Brain water content measurement

Mice were euthanized and their brains were rapidly collected. The contralateral and ipsilateral hemispheres as well as cerebellum were used for analysis. After obtaining the wet weights using an analytical balance, collected tissues were dried for 24 h and weighed again (dry weight). Brain water content was calculated as follows: brain water content = (wet weight − dry weight)/wet weight × 100 [30, 39].

### BBB permeability assay

BBB permeability was examined using in vivo permeability assay as described previously [40, 41]. Briefly, sterile Evans blue solution (2% in saline, 80 μl) and FITC-Dextran (4 kD, 25 mg/ml, 50 μl) were injected intravenously into control and PKO mice. After 6 h, these mice were transcardially perfused with 50 ml saline. The brains were then collected and cut into left and right hemispheres. Each hemisphere was carefully weighed and homogenized in 800 μl PBS and centrifuged at 16,363 g (Eppendorf FA-45-24-11 rotor) for 20 min at 4 °C. For Evans blue, the supernatant was collected and read in a spectrophotometer (Molecular devices-SpectraMax, California, USA) at 620 nm. For FITC-Dextran, the supernatant was collected and read in a fluorescent plate reader (Molecular devices-SpectraMax, California, USA) at 485/528 nm. Each sample was measured in triplicates, and the average of these technical replicates was used as one biological replicate.

### Transmission electron microscopy (TEM)

Mice were anesthetized and perfused with PBS followed by 0.1-M sodium cacodylate buffer containing 2% PFA and 2% glutaraldehyde. Brain tissue from the peri-hematoma zone was dissected out, fixed overnight, and post-fixed in 1% osmium tetroxide and 1% K-ferrocyanide. Next, the tissue was en bloc stained with 2% uranyl acetate and embedded in resin. Ultra-thin sections were cut on an RMC MT-X microtome (Boeckeler Instruments) and post-stained with 2% uranyl acetate and 1% lead citrate. Sections were examined and photographed using JEOL JEM1011 (JEOL) at 80 kV.

### Cell culture

Mouse brain endothelial cells (bEnd.3, CRL-2299), mouse bone marrow cells (LADMAC, CRL-2420), and mouse brain microglia (EOC 13.31, CRL-2468) were purchased from ATCC. bEnd.3 cells were cultured in standard medium [Dulbecco’s modified Eagle’s medium (DMEM) supplemented with 10% fetal bovine serum (FBS), 100 units/ml penicillin and 100 μg/ml streptomycin]. LADMAC cells were cultured in Eagle’s minimum essential medium (MEM) supplemented with 10% FBS, 100 units/ml penicillin, and 100 μg/ml streptomycin. EOC 13.31 cells were cultured in standard medium supplemented with 20% LADMAC conditioned media. All cells were cultured at 37 °C under a 5% CO_2_ atmosphere.

### In vitro ICH model

The in vitro ICH model was established as described previously [42]. Briefly, EOC 13.31 cells were treated with 10-μM hemoglobin for 24 h and the supernatant was collected as microglia-conditioned medium. When reached confluence, bEnd.3 cells were treated with the collected microglia-conditioned medium to mimic ICH in vitro. This experiment was performed in the presence or absence of 10 μg/ml laminin-421/-521 (Biolamina). After 24 h, bEnd.3 cells were collected for western blot analysis.

### Western blotting

Cells were lysed with RIPA buffer (50-mM Tris pH 7.4, 1% NP-40, 0.5% Na-deoxycholate, 1% SDS, 150-mM NaCl, 2-mM EDTA, 1 × protease inhibitor cocktail, and 1 × phosphatase inhibitor cocktail). Total protein levels were determined using the Bio-Rad protein assay kit, and equal amounts of proteins were loaded and separated on SDS-PAGE. After transferring to PVDF membrane (Millipore), proteins were detected using a standard immune-blotting technique. The following primary antibodies were used: mouse anti-claudin-5 (1:500, Invitrogen, USA, 35-2500), rabbit anti-ZO-1 (1:500, Thermofisher, USA, 61-7300), rabbit anti-caveolin-1 (1:1000, cell signaling, 3238S), and mouse anti-β-actin (Sigma, A5441, 1:2000). Target proteins were visualized using SuperSignal West Pico Plus Chemiluminescent Substrate (Thermo scientific). The density of target protein bands was quantified using NIH ImageJ software. The expression of target proteins was normalized to that of β-actin.

### Statistics

Prism 6 (GraphPad Software) was used to perform statistical analyses. Student’s *t* test was used to analyze differences between two groups. One-way ANOVA followed by Neuman Keuls post hoc analysis was used when more than two groups were compared. *p < 0.05* was considered to be significant. Sample number (*n*) represents biological replicates. Sample sizes for experiments were shown in the figure legends. Results are shown as mean ± SD.

## Results

### PKO mice show enlarged hematoma after ICH

**Fig. 1:**
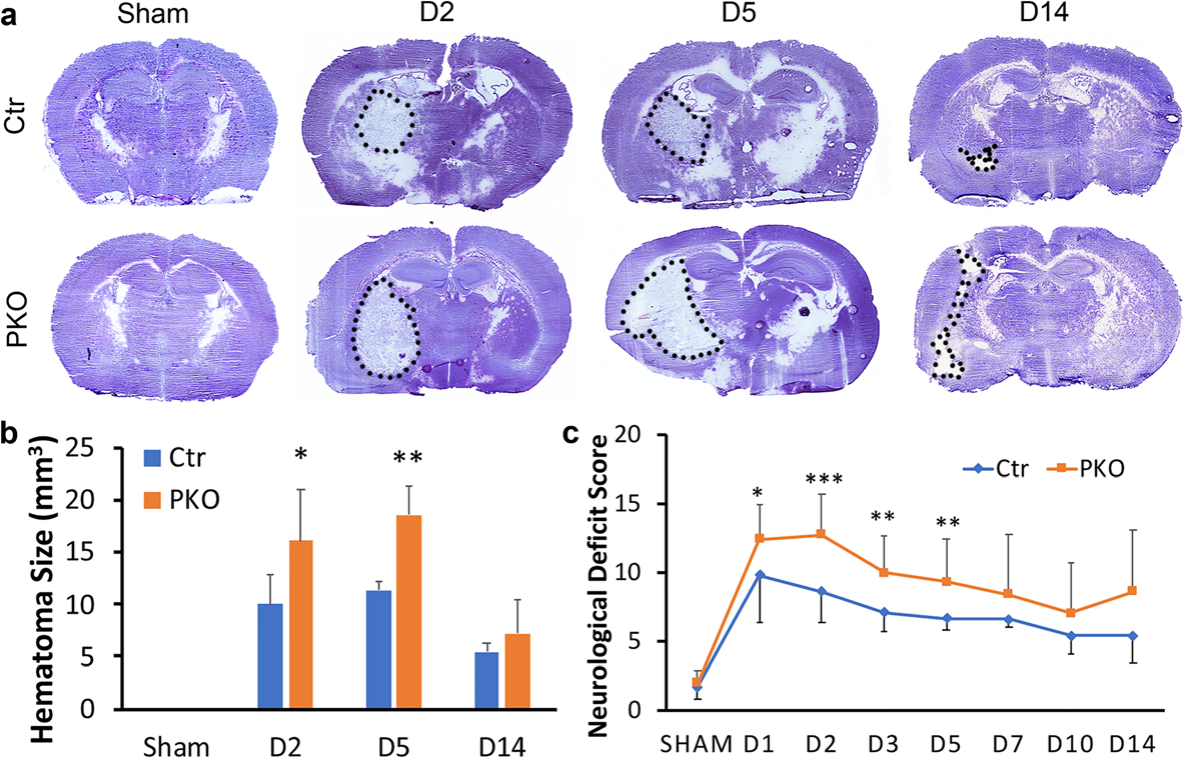
PKO mice show enlarged hematoma size and increased neurological deficit score after ICH. **a** Representative images of hematoxylin-stained control and PKO brains from sham group and at D2, D5, and D14 after ICH. Dashed lines mark injury areas. **b** Quantification of injury volume in control and PKO mice. *p < 0.05 and **p < 0.01, compared to controls at the same time points (Student’s *t* test), *n* = 6. **c** Neurological deficit score in control and PKO mice from sham group and at D1, D2, D3, D5, D7, D10, and D14 after ICH. *p < 0.05 and **p < 0.01, ***p < 0.001, compared to controls at the same time points (Student’s *t* test), *n* = 5–15 (*n* = 15 for D1–D2, *n* = 12–14 for D3–D5, and *n* = 5 for sham and D7–D14). Data are shown as mean ± SD

Hematoma volume is one of the major determinants of mortality and morbidity in ICH outcomes [43]. Although no hematoma was found in sham animals, both control and PKO mice showed hematoma after ICH (Fig. 1a). Control mice showed a hematoma size of 10.01 ± 2.91 mm^3^ at day 2 after injury (Fig. 1a, b). Hematoma size remained similar at day 5 after injury (11.39 ± 0.81 mm^3^) and substantially reduced by day 14 after injury (5.41 ± 0.83 mm^3^) (Fig. 1a, b). Although a similar trend was observed in PKO mice, these mutants displayed increased hematoma volume at all three time points (16.14 ± 4.96 mm^3^, 18.59 ± 2.80 mm^3^, 7.22 ± 3.27 mm^3^) compared to the controls (Fig. 1a, b). Statistical significance, however, was only achieved at day 2 and day 5 after injury. These results suggest that mural cell-derived laminin negatively regulates hematoma size.

### PKO mice show worse neurological function after ICH

Focal neurological deficits occur after ICH [44, 45]. To assess neurological function, we scored control and PKO mice at various time points after injury using a well-established scoring system [30, 36]. Sham animals, independent of genotypes, showed very low neurological deficit scores, suggesting normal neurological function (Fig. 1c). Consistent with previous reports [30, 36], control mice showed a high neurological deficit score at day 1 after injury (9.80 ± 3.46), which slowly decreased overtime (8.60 ± 2.23, 7.08 ± 1.38, 6.67 ± 0.89, 6.60 ± 0.55, 5.40 ± 1.34, and 5.40 ± 1.95 in day 2, day 3, day 5, day 7, day 10, and day 14, respectively) (Fig. 1c). Like in the controls, neurological deficit score decreased overtime after ICH in PKO mice (12.40 ± 2.53, 12.73 ± 2.91, 9.93 ± 2.76, 9.29 ± 3.10, 8.40 ± 4.39, 7.00 ± 3.74, and 8.60 ± 4.51 in day 1, day 2, day 3, day 5, day 7, day 10, and day 14, respectively) (Fig. 1c). Compared to the controls, the PKO mice displayed higher neurological deficit scores at all time points examined, although statistical significance was only achieved in early time points (up to day 5 after injury) (Fig. 1c). These findings suggest that mural cell-derived laminin plays a beneficial role in ICH.

### PKO mice show enhanced neuronal death after ICH

**Fig. 2:**
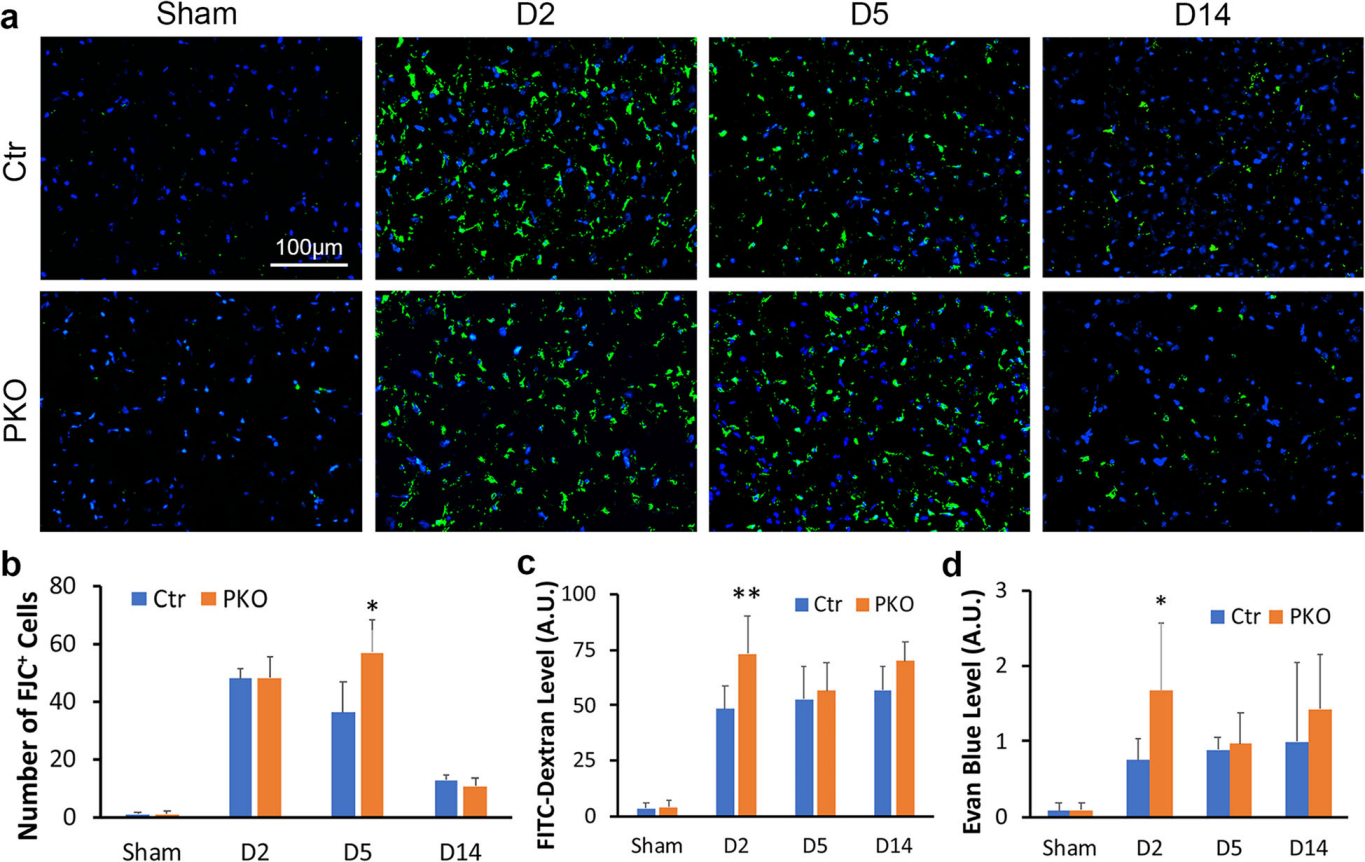
PKO mice show enhanced neuronal cell death and exacerbated BBB disruption after ICH. **a** Representative images of FJC-stained control and PKO brains from sham group and at D2, D5, and D14 after ICH. **b** Quantification of FJC^+^ cells in control and PKO mice. *p < 0.05, compared to controls at the same time points (Student’s *t* test), *n* = 3–4. **c**, **d** Quantifications of FITC-Dextran (**c**) and Evans blue (**d**) leakage in control and PKO brains from sham group and at D2, D5, and D14 after ICH. *p < 0.05, compared to controls at the same time points (ANOVA followed by Newman-Keuls test), *n* = 8–14 in (**c**) and *n* = 6–11 in (**d**). Data are shown as mean ± SD

To determine if loss of mural cell-derived laminin affects neuronal death after ICH, we performed FJC staining, which marks degenerating neurons [30, 37]. As expected, we failed to detect FJC^+^ cells in sham animals independent of genotypes (Fig. 2a). Control (48.10 ± 3.62) and PKO (48.40 ± 7.22) mice demonstrated comparable FJC^+^ cells at day 2 after ICH (Fig. 2a, b). Compared to the controls, significantly more FJC^+^ cells were found in PKO brains at day 5 (36.43 ± 10.79 in control and 57.18 ± 11.21 in PKO) but not day 14 (13.13 ± 1.75 in control and 11.00 ± 2.85 in PKO) after injury (Fig. 2a, b), again suggesting aggravated injury in the mutants.

### PKO mice show exacerbated BBB disruption after ICH

BBB disruption is a hallmark of ICH-induced brain injury [46, 47]. To determine BBB permeability, we intravenously injected 4kD-FITC-Dextran into control and PKO mice and examined its leakage into brain parenchyma at 2, 5, and 14 days after ICH. Consistent with our previous finding that BBB integrity is intact in PKO mice at young age [28], FITC-Dextran leakage was not found in sham animals independent of genotypes (Fig. 2c). Although increased FITC-Dextran level was found in PKO brains at all three time points after ICH, statistical significance was only achieved at day 2 (48.28 ± 10.36 in control and 73.25 ± 17.06 in PKO) after injury (Fig. 2c). To determine the extent of BBB disruption, Evans blue, which serves as a ~ 66kD tracer due to albumin binding, was also used. Again, no leakage of Evan blue was detected in sham animals (Fig. 2d). Similar to FITC-Dextran, a significantly higher level of Evan blue was found in PKO brains (1.67 ± 0.90) at day 2 after injury compared to the controls (0.75 ± 0.29) (Fig. 2d). Although a trend was detected at day 5 and day 14 after injury, statistical significance was not reached at these time points. These findings suggest that mural cell-derived laminin contributes to BBB recovery at early stage after ICH.

### Loss of mural cell-derived laminin does not affect paracellular transport after ICH

**Fig. 3:**
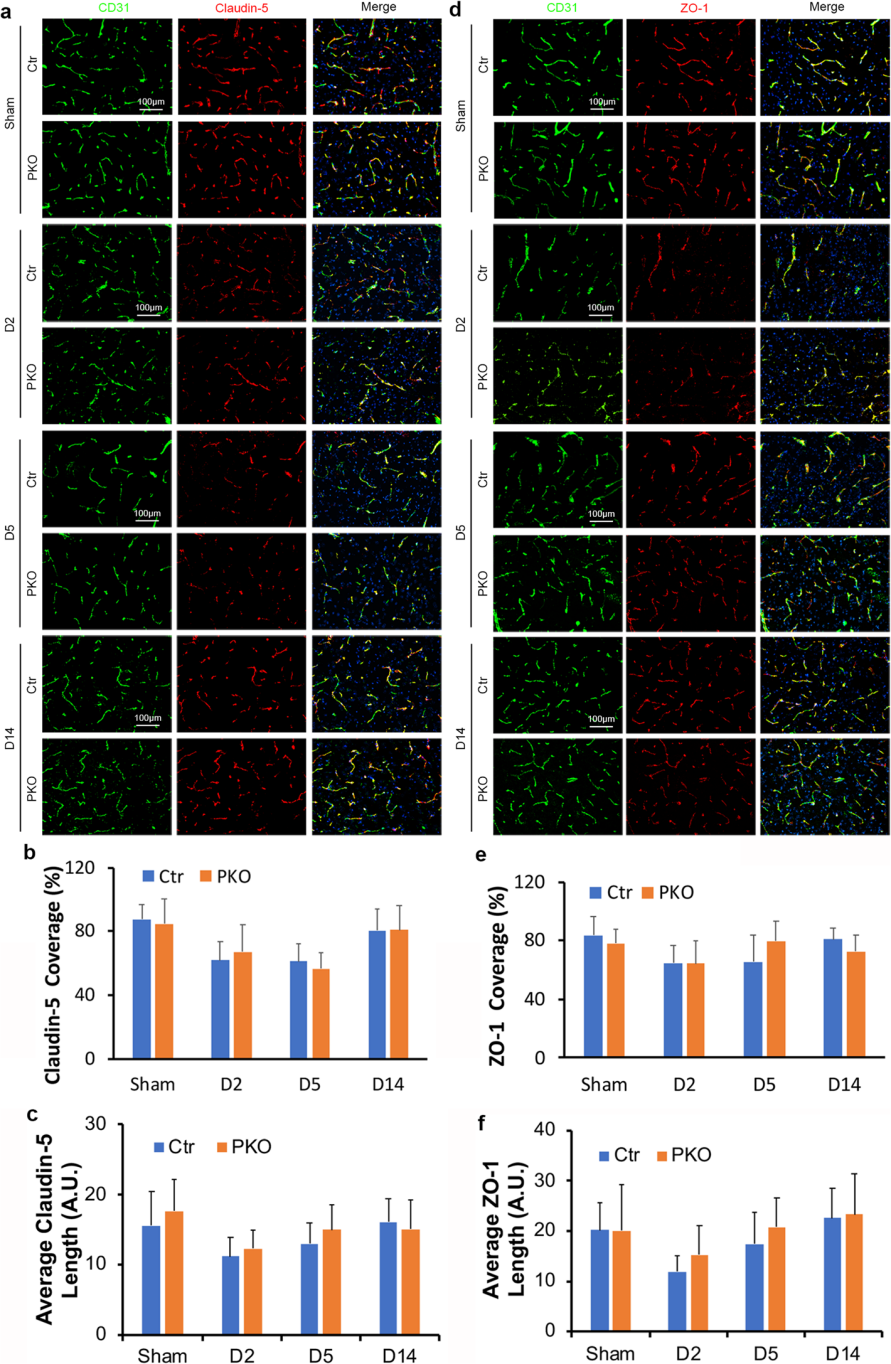
Loss of mural cell-derived laminin does not affect paracellular transport after ICH. **a** Immunohistochemistry of CD31 (green)/claudin-5 (red) in control and PKO brains from sham group and D2, D5, and D14 after ICH. **b** Quantification of claudin-5 coverage in control and PKO brains. *n* = 5. **c** Quantification of average claudin-5 length in control and PKO brains. *n* = 5. **d** Immunohistochemistry of CD31 (green)/ZO-1 (red) in control and PKO brains from sham group and D2, D5, and D14 after ICH. **e** Quantification of ZO-1 coverage in control and PKO brains. *n* = 5. **f** Quantification of average ZO-1 length in control and PKO brains. *n* = 5. Data are shown as mean ± SD

Tight junction proteins (TJPs) play an important role in maintaining paracellular barrier of the BBB [48–50]. To determine if decreased TJP is responsible for the enhanced BBB disruption in PKO mice after ICH, we performed co-staining of vascular marker CD31 and two TJPs (claudin-5 and ZO-1). Consistent with our previous finding that TJP expression is unaffected in PKO mice at young age [28], sham mice showed comparable and high levels of claudin-5 coverage between genotypes (87.35 ± 9.31 for control and 84.45 ± 16.20 for PKO) (Fig. 3a, b). After ICH, control mice demonstrated reduced claudin-5 coverage at day 2 (61.76 ± 11.56), day 5 (60.81 ± 11.53), and day 14 (80.49 ± 13.23) (Fig. 3a, b). Similar changes on claudin-5 coverage were observed in PKO mice at day 2 (67.04 ± 16.81), day 5 (56.33 ± 10.13), and day 14 (80.55 ± 15.54) after injury (Fig. 3a, b). Comparison between genotypes, however, failed to reveal statistical significance at any time point (Fig. 3b). In addition to claudin-5 coverage, we also quantified average claudin-5 length (Fig. 3c) between genotypes and found no statistical significance.

Like claudin-5, ZO-1 displayed high coverage in sham mice (83.46 ± 12.89 for control and 78.09 ± 9.76 for PKO) (Fig. 3d, e). After ICH, ZO-1 coverage decreased in both control (64.67 ± 12.14, 65.43 ± 18.22, and 80.84 ± 7.37 at day 2, day 5, and day 14 after injury, respectively) and PKO (64.12 ± 15.84, 79.78 ± 13.61, and 72.55 ± 10.85 at day 2, day 5, and day 14 after injury, respectively) mice (Fig. 3d, e). No statistical significance was detected between genotypes (Fig. 3e). Similarly, average ZO-1 length (Fig. 3f) failed to show a significant difference between genotypes at any time point.

**Fig. 4:**
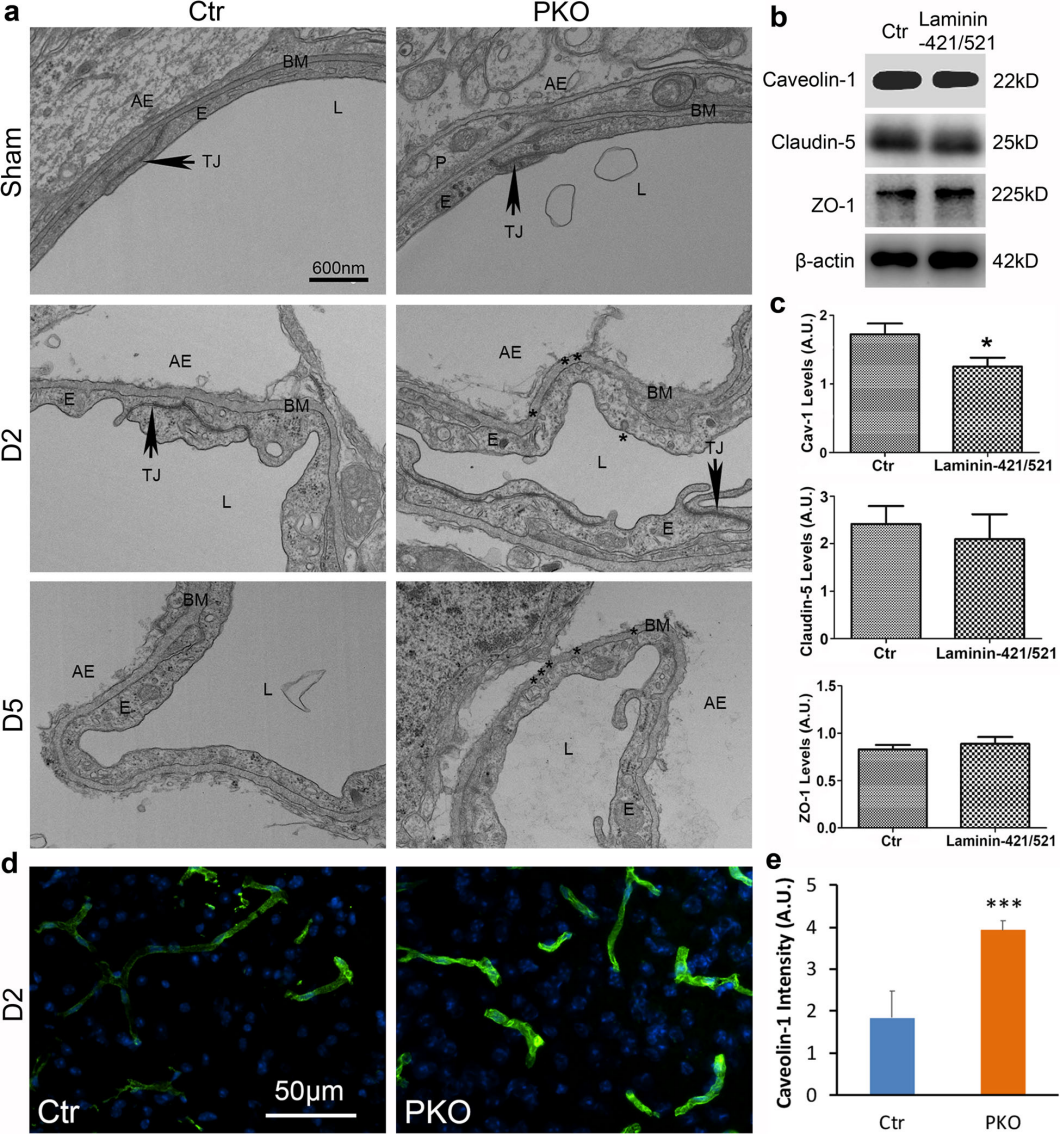
Loss of mural cell-derived laminin exacerbates transcellular transport after ICH. **a** TEM images showing the ultrastructure of the BBB in control and PKO mice from sham group or at day 2 and day 5 after ICH. Arrows indicate tight junctions and stars indicate pinocytotic vesicles. AE, astrocytic endfeet; BM, basement membrane; E, endothelial cell; L, lumen; P, pericyte; TJ, tight junction. **b**, **c** Representative western blots (**b**) and quantifications (**c**) of caveolin-1, claudin-5, and ZO-1 expression in endothelial cells after in vitro ICH. *p < 0.05, compared to controls (Student’s *t* test), *n* = 6. **d** Representative images of caveolin-1 staining in control and PKO brains at day 2 after ICH. **e** Quantification of caveolin-1 intensity in control and PKO brains at day 2 after ICH. ***p < 0.001, compared to the control (Student’s *t* test), *n* = 3. Data are shown as mean ± SD

Next, we further examined the morphology of tight junctions at the ultrastructural level using TEM. In sham animals, both genotypes displayed nicely aligned electron-dense tight junctions at cell-cell borders (Fig. 4). After ICH, tight junctions became irregular, detached, or even lost in some regions (Fig. 4a). Comparison between genotypes, however, failed to reveal any difference (Fig. 4a). Together, these findings suggest that mural cell-derived laminin does not affect paracellular transport in endothelial cells after ICH.

### Loss of mural cell-derived laminin increases transcellular transport after ICH

In addition to intercellular tight junctions, extremely low transcellular transport also contributes to BBB integrity [51–53]. To determine if increased transcellular transport is responsible for the enhanced BBB leakage in PKO mice after ICH, we examined transcytosis by TEM. In sham controls, few pinocytotic vesicles and a thin layer of basement membrane (BM) were observed independent of the genotypes (Fig. 4a), indicating unaltered transcellular transport in PKO brains under homeostatic conditions. After ICH, we observed swollen astrocytic endfeet and thickened BM in both genotypes (Fig. 4a), indicating successful induction of injury. Interestingly, significantly more endothelial pinocytotic vesicles were found in PKO mice at day 2 and day 5 after ICH, compared to the controls (Fig. 4a), indicating enhanced transcellular transport in PKO brains. These results suggest that mural cell-derived laminin negatively regulates transcellular transport in endothelial cells after ICH.

### Mural cell-derived laminin inhibits transcytosis by downregulating caveolin-1

To explore the molecular mechanism underlying mural cell-derived laminin’s effect on transcytosis in endothelial cells after ICH, we moved to a simplified in vitro ICH model that involves hemoglobin and microglia-conditioned medium [42]. Previous studies have shown that caveolae-mediated transcytosis plays an important role in BBB permeability regulation [51–53]. To determine if mural cell-derived laminin regulates transcytosis by targeting this pathway, we examined the expression of caveolin-1, a protein indispensable for caveolae-mediated transcytosis [54, 55]. We found that exogenous mural cell-derived laminin (laminin-421/521) significantly reduced caveolin-1 expression without affecting the levels of claudin-5 and ZO-1 in bEnd.3 cells in this in vitro ICH model (Fig. 4b, c), suggesting that mural cell-derived laminin inhibits transcytosis by downregulating caveolin-1 in endothelial cells after ICH.

To further investigate the role of mural cell-derived laminin in caveolin-1 expression in vivo, we examined caveolin-1 expression in control and PKO brains at day 2 after ICH, a time point when enhanced BBB leakage was observed in PKO mice (Fig. 2c, d). Consistent with previous reports that caveolin-1 is predominantly expressed in endothelial cells in the brain [56–59], caveolin-1 displayed a blood vessel-like pattern in the brain (Fig. 4d). Compared to the controls, caveolin-1 fluorescent intensity was significantly increased in PKO brains at day 2 after ICH (Fig. 4d, e), suggesting that mural cell-derived laminin negatively regulates caveolin-1 expression in brain endothelial cells after ICH. Altogether, these findings suggest that mural cell-derived laminin inhibits transcytosis by downregulating caveolin-1 in brain endothelial cells after ICH.

### Loss of mural cell-derived laminin fails to affect pericyte coverage after ICH

**Fig. 5:**
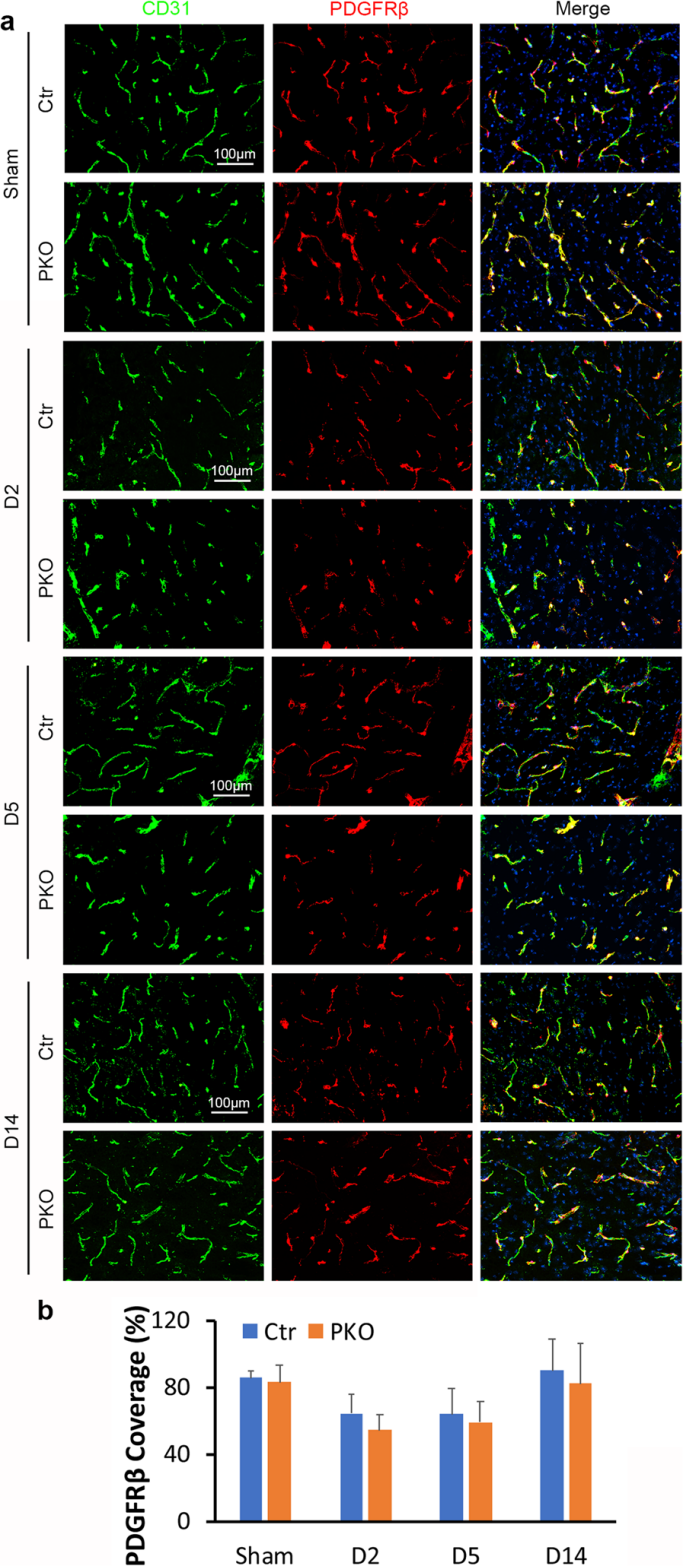
Loss of mural cell-derived laminin fails to affect pericyte coverage after ICH. **a** Immunohistochemistry of CD31 (green)/PDGFRβ (red) in control and PKO brains from sham group and D2, D5, and D14 after ICH. **b** Quantification of pericyte coverage in control and PKO brains. *n* = 5. Data are shown as mean ± SD

Accumulating evidence suggests that pericyte coverage actively contributes to BBB maintenance [60–62]. To determine if reduced pericyte coverage contributes to the elevated BBB permeability in PKO mice after ICH, we performed immunohistochemistry against vascular marker CD31 and pericyte marker PDGFRβ. Consistent with our previous finding that pericyte coverage is unchanged in PKO mice at young age [28], sham mice showed comparable and high levels of pericyte coverage between genotypes (85.66 ± 4.19 for control and 82.85 ± 10.07 for PKO) (Fig. 5a). After ICH, control mice showed an initial reduction of pericyte coverage at day 2 (64.22 ± 11.89) and day 5 (64.06 ± 15.36), and a subsequent increase of pericyte coverage by day 14 (90.10 ± 18.77) (Fig. 5a, b). Pericyte coverage changes in PKO mutants (54.49 ± 9.26, 59.04 ± 12.06, and 82.25 ± 23.96 at day 2, day 5, and day 14 after injury, respectively) mimicked those in control mice (Fig. 5a, b). Comparison between genotypes revealed no statistical significance at any time point (Fig. 5b). These results suggest that loss of mural cell-derived laminin does not affect pericyte coverage after ICH.

### PKO mice show enhanced inflammatory cell extravasation after ICH

**Fig. 6:**
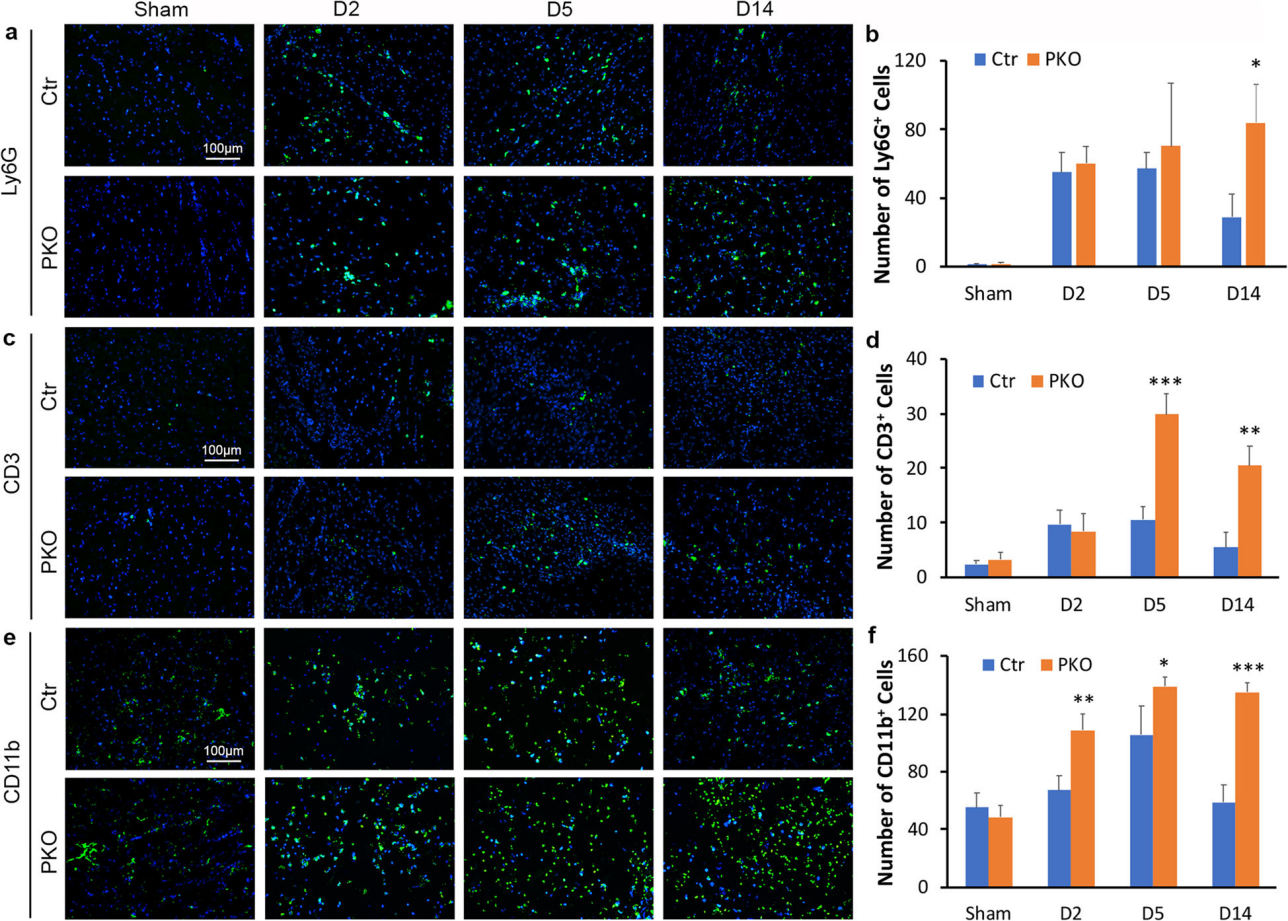
PKO mice show enhanced inflammatory cell extravasation after ICH. **a** Immunohistochemistry of Ly6G in control and PKO brains from sham group and at D2, D5, and D14 after ICH. **b** Quantification of Ly6G^+^ neutrophils in control and PKO mice. *p < 0.05, compared to controls at the same time points (ANOVA followed by Newman-Keuls test), *n* = 3–4. **c** Immunohistochemistry of CD3 in control and PKO brains from sham group and at D2, D5, and D14 after ICH. **d** Quantification of CD3^+^ T cells in control and PKO mice. **p < 0.01 and ***p < 0.001, compared to controls at the same time points (ANOVA followed by Newman-Keuls test), *n* = 3–4. **e** Immunohistochemistry of CD11b in control and PKO brains from sham group and at D2, D5, and D14 after ICH. **f** Quantification of CD11b^+^ microglia/macrophages in control and PKO mice. *p < 0.05, **p < 0.01, and ***p < 0.001, compared to controls at the same time points (ANOVA followed by Newman-Keuls test), *n* = 3–4. Data are shown as mean ± SD

Inflammatory cells infiltrate into brain parenchyma after ICH. To determine if this process is affected by loss of mural cell-derived laminin, we examined inflammatory cell extravasation by immunohistochemistry. First, we examined neutrophil infiltration using Ly6G. Few Ly6G^+^ cells were detected in sham animals (Fig. 6a). In control mice, the numbers of Ly6G^+^ neutrophils were high at day 2 (54.88 ± 12.01) and day 5 (57.01 ± 9.64) after injury, but low at day 14 (28.70 ± 13.79) after injury (Fig. 6a, b). Unlike control mice, PKO mice showed a continuous increase of Ly6G^+^ neutrophils after ICH (60.35 ± 9.90, 70.40 ± 37.06, and 83.96 ± 22.31 at day 2, day 5, and day 14 after injury, respectively) (Fig. 6a, b). Comparison between genotypes revealed statistical significance at day 14 after injury, indicating increased extravasation of neutrophils in PKO brains.

Next, we examined T cell extravasation using CD3. Similarly, CD3^+^ cells were barely detected in sham animals (Fig. 6c). In control mice, T cells were slightly increased at injury (9.67 ± 2.57, 10.58 ± 2.34, and 5.57 ± 2.79 at day 2, day 5, and day 14 after injury, respectively) (Fig. 6c, d). Compared to the controls, significantly more CD3^+^ cells were detected in PKO mice at day 5 (29.83 ± 3.89) and day 14 (20.45 ± 3.62) after injury (Fig. 6c, d), suggesting enhanced T cell infiltration.

Furthermore, we examined monocyte/macrophage extravasation using CD11b. Consistent with previous reports that CD11b is able to label brain-resident microglia in addition to monocytes/macrophages [63, 64], CD11b^+^ cells (microglia) were detected in sham animals (Fig. 6e). Quantification of CD11b^+^ cells showed no statistical significance between genotypes in sham animals (55.33 ± 10.25 in control and 48.29 ± 8.04 in PKO) (Fig. 6f), suggesting a dispensable role of mural cell-derived laminin in microglial number without ICH. After ICH, the number of CD11b^+^ cells peaked at day 5 (105.47 ± 20.29) after injury and decreased by day 14 (58.68 ± 12.03) after injury in control mice (Fig. 6e, f). Although CD11b^+^ cells peaked at day 5 (138.78 ± 6.78) after injury in PKO mice, they remained high at day 14 (134.86 ± 6.90) after injury (Fig. 6e, f). Compared to controls, PKO mice demonstrated significantly more CD11b^+^ cells at all three time points examined (Fig. 6f), suggesting increased monocyte/macrophage extravasation. Together, these findings strongly suggest that mural cell-derived laminin negatively regulates inflammatory cell infiltration after ICH.

### PKO mice show abnormal microglial activation at the recovery phase after ICH

**Fig. 7:**
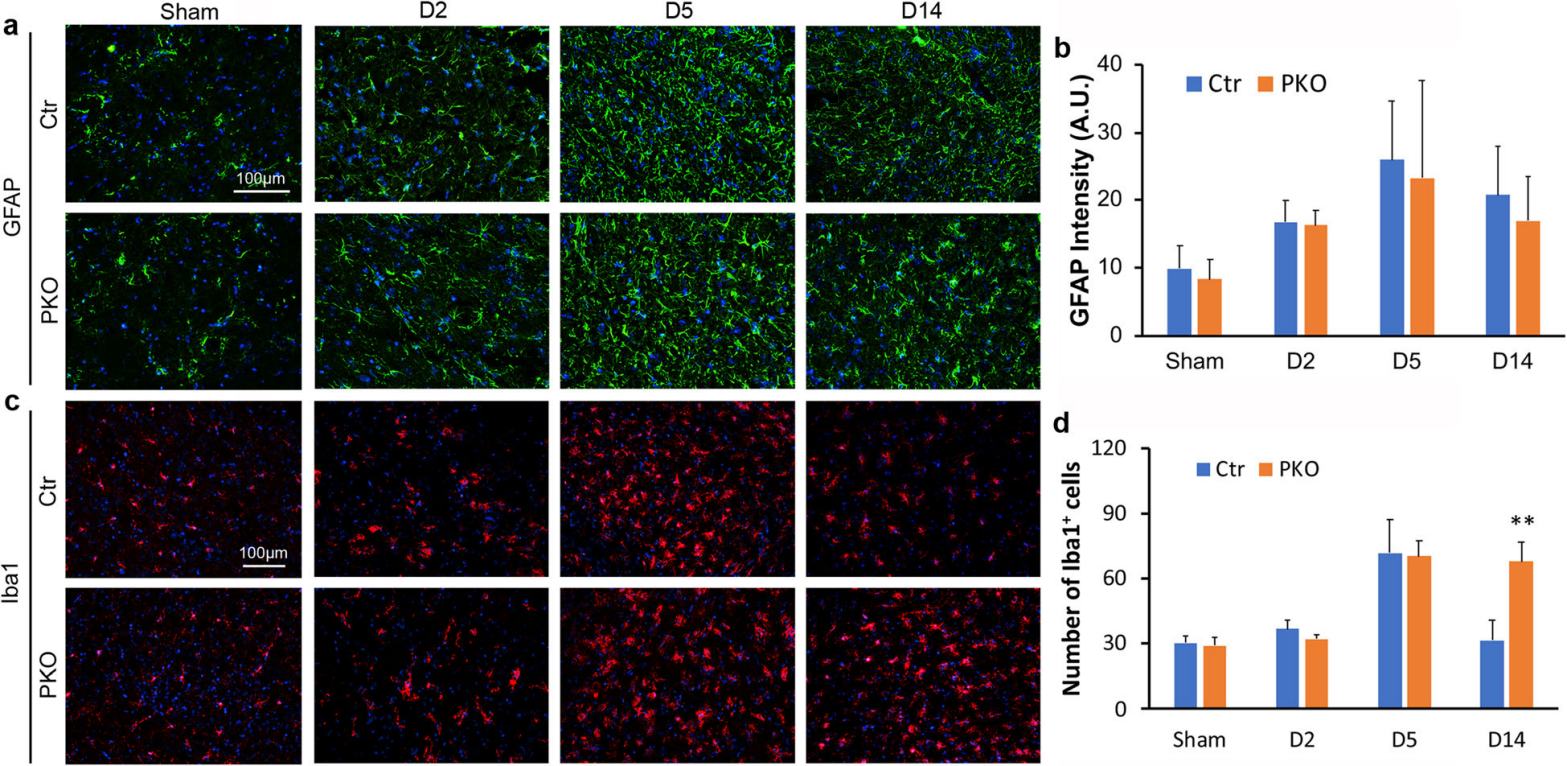
PKO mice show no changes in astrogliosis but aggravated microglial activation after ICH. **a** Immunohistochemistry of GFAP in control and PKO brains from sham group and at D2, D5, and D14 after ICH. **b** Quantification of GFAP intensity in control and PKO mice. *n* = 3. **c** Immunohistochemistry of Iba-1^+^ cells in control and PKO brains from sham group and at D2, D5, and D14 after ICH. **d** Quantification of Iba-1^+^ cells in control and PKO mice. **p < 0.01, compared to controls at the same time points (ANOVA followed by Newman-Keuls test), *n* = 3. Data are shown as mean ± SD

ICH induces reactive astrogliosis and microglial activation [65–67]. To determine if loss of mural cell-derived laminin affects these processes, reactive astrogliosis and microglial activation were examined by GFAP and Iba1 staining, respectively. Weak GFAP signal was found in sham animals (Fig. 7a). Quantification revealed comparable GFAP intensity in sham control (9.80 ± 3.41) and sham PKO (8.29 ± 2.87) mice (Fig. 7b). After ICH, GFAP signal increased at day 2 (16.76 ± 3.19), peaked at day 5 (25.97 ± 8.70), and slightly decreased at day 14 (20.76 ± 7.17) in control mice (Fig. 7a, b). A similar pattern was observed in PKO mice (16.30 ± 2.22, 23.30 ± 14.32, and 16.95 ± 6.62, at day 2, day 5, and day 14 after injury, respectively) (Fig. 7a, b). Quantification showed that GFAP levels were comparable between genotypes at each time point, suggesting that loss of mural cell-derived laminin does not affect astrogliosis after ICH.

Iba1^+^ cells displayed ramified morphology (microglia) in sham animals (Fig. 7c). Quantification revealed comparable numbers of Iba1^+^ cells in sham control (30.20 ± 3.56) and sham PKO (29.00 ± 3.94) mice (Fig. 7d), suggesting a dispensable role of mural cell-derived laminin in microglial number under homeostatic condition. After ICH, Iba1^+^ cells changed to an ameboid morphology in both control and PKO mice (Fig. 7c), indicating microglial activation. In control mice, the number of Iba1^+^ microglia peaked at day 5 after injury (71.85 ± 15.73) and decreased at day 14 after injury (31.59 ± 9.01) (Fig. 7d). Unlike in control mice, Iba1^+^ microglia number remained high at day 14 after injury (68.04 ± 8.93) in PKO mice (Fig. 7d). Quantification revealed significantly increased Iba1^+^ microglia in PKO brains at day 14 after injury but not at earlier time points (Fig. 7d). These results suggest that mural cell-derived laminin negatively regulate microglial number at the recovery phase after ICH.

### PKO mice show elevated brain water content before and after ICH

Red blood cell lysis, hemoglobin toxicity, BBB disruption, and inflammatory cell extravasation contribute to edema formation after ICH [68, 69]. To determine if lack of mural cell-derived laminin exacerbates brain edema, we measured brain water content in control and PKO mice at various time points after ICH. Compared to controls, PKO mice showed significantly higher brain water content in the ipsilateral hemisphere at day 5 (79.07 ± 0.40 in control and 80.71 ± 0.86 in PKO), but not at day 2 (79.78 ± 0.90 in control and 79.87 ± 0.33 in PKO) or day 14 (78.28 ± 0.34 in control and 78.72 ± 0.62 in PKO) after injury (Table 1), suggesting an important role of mural cell-derived laminin in brain water homeostasis after ICH.

**Table 1:** Brain water content in control and PKO mice

Genotype	Group	Brain water content (%)		
		Contralateral	Ipsilateral	Cerebellum
Control	Sham	78.56 ± 0.24	78.50 ± 0.22	77.33 ± 0.37
	D2	78.64 ± 0.58	79.78 ± 0.90^##^	76.95 ± 0.51
	D5	78.59 ± 0.30	79.07 ± 0.40	76.98 ± 0.52
	D14	77.95 ± 0.39	78.28 ± 0.34	76.93 ± 0.52
PKO	Sham	79.80 ± 0.18**	79.72 ± 0.43**	79.01 ± 0.60**
	D2	79.50 ± 0.32	79.87 ± 0.33	78.67 ± 0.40
	D5	79.97 ± 0.53	80.71 ± 0.86**	78.49 ± 0.25
	D14	78.73 ± 0.49	78.72 ± 0.62	77.67 ± 0.89

^##^*p* < 0.01, compared to contralateral hemisphere within the same genotype; **p < 0.01, compared to wildtype controls. Student’s *t* test. *n* = 4–10

In addition, we also compared brain water content between contralateral and ipsilateral hemispheres within each genotype. In control mice, the ipsilateral hemisphere showed significantly higher brain water content at day 2 after ICH (79.78 ± 0.90), which slowly went down overtime (79.07 ± 0.40 and 78.28 ± 0.34 at day 5 and day 14 after injury, respectively) (Table 1), indicating successful induction of ICH. In PKO mice, however, the contralateral and ipsilateral hemispheres displayed comparable brain water content (Table 1). This is mainly due to the high baseline brain water content in the contralateral hemisphere in PKO mice (78.64 ± 0.58, 78.59 ± 0.30, and 77.95 ± 0.39 in control vs. 79.50 ± 0.32, 79.97 ± 0.53, and 78.73 ± 0.49 in PKO at day 2, day 5, and day 14 after ICH, respectively) (Table 1). Similar changes were observed in the cerebellum (76.95 ± 0.51, 76.98 ± 0.52, and 76.93 ± 0.52 in control vs. 78.67 ± 0.40, 78.49 ± 0.25, and 77.67 ± 0.89 in PKO at day 2, day 5, and day 14 after ICH, respectively) (Table 1).

To determine if the enhanced baseline brain water content in PKO mice is intrinsic to the mutants or induced by ICH, we further examined brain water content in sham animals. Compared to the controls, PKO mice demonstrated significantly higher levels of brain water content in contralateral hemisphere (78.56 ± 0.24 in control vs. 79.80 ± 0.18 in PKO), ipsilateral hemisphere (78.50 ± 0.22 in control vs. 79.72 ± 0.43 in PKO), and cerebellum (77.33 ± 0.37 in control vs. 79.01 ± 0.60 in PKO) (Table 1). These results suggest an indispensable role of mural cell-derived laminin in brain water homeostasis independent of ICH.

### AQP4 level is unaffected in PKO mice

**Fig. 8:**
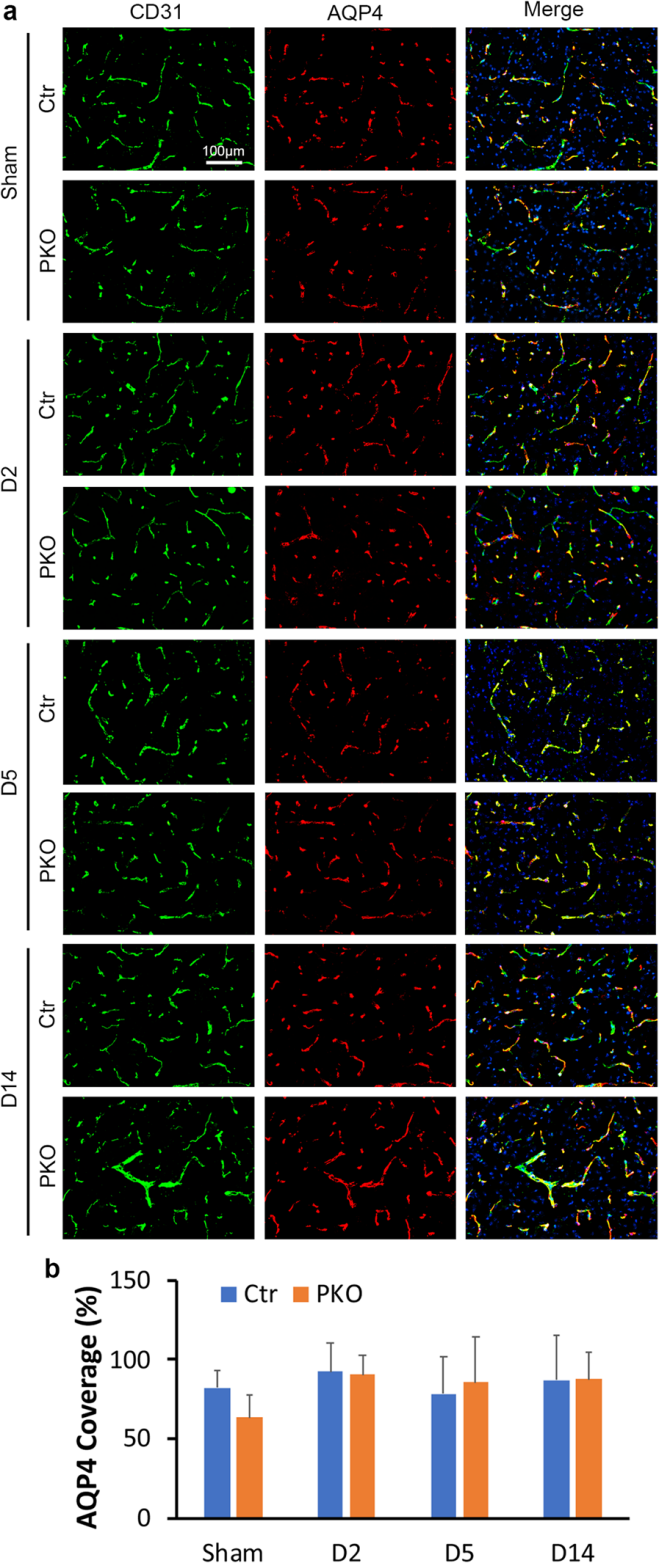
Loss of mural cell-derived laminin does not affect AQP4 level. **a** Immunohistochemistry of AQP4 (green)/CD31 (red) in control and PKO brains from sham groups and at D2, D5, and D14 after ICH. **b** Quantification of AQP4 coverage in control and PKO mice. *n* = 4. Data are shown as mean ± SD

As an important water channel expressed exclusively at astrocytic endfeet, AQP4 actively regulates water homeostasis in the brain [70, 71]. To determine if the enhanced brain water content in PKO mice is caused by abnormal AQP4 expression, we performed immunohistochemistry against AQP4 and vascular marker CD31 (Fig. 8a). Consistent with our previous report [28], comparable AQP4 coverage (82.19 ± 11.31 in control and 63.23 ± 14.33 in PKO) were found in sham animals (Fig. 8a, b). Similarly, no difference in AQP4 coverage was observed between genotypes at any time point after ICH (92.11 ± 18.39, 78.34 ± 23.60, and 87.03 ± 28.03 in control vs. 90.19 ± 13.02, 85.49 ± 28.97, and 87.28 ± 17.25 in PKO at day 2, day 5, and day 14 after ICH, respectively) (Fig. 8a, b). These results suggest that the abnormally high brain water content in PKO mice is AQP4-independent.

## Discussion

In this study, we examined brain injury in PKO mice using collagenase-induced ICH model. The PKO mice displayed worse outcomes after ICH, including enlarged hematoma volume, aggravated neurological dysfunction, increased neuronal degeneration, exacerbated BBB disruption, enhanced inflammatory cell infiltration, and elevated microglial activation, suggesting that mural cell-derived laminin plays a beneficial role after ICH. We further demonstrated that mural cell-derived laminin maintains BBB integrity by downregulating caveolin-1 and thus transcytosis in endothelial cells after ICH.

The functional significance of laminin in ICH is largely unknown. Previous correlation studies revealed reduced laminin levels in ICH brains and in hemorrhagic areas of ischemic brains [72, 73], although the specific laminin isoforms reduced were not reported, suggesting a possibly neuroprotective role of laminin in ICH. Consistent with this speculation, we demonstrated that mice lacking astrocytic laminin developed severe BBB disruption and age-dependent spontaneous ICH [20, 25], suggesting a causative role of astrocytic laminin in ICH pathogenesis. In addition, in a recent study, we reported that endothelium-specific laminin-α5 conditional knockout mice were grossly normal under homeostatic condition but showed exacerbated brain injury in collagenase-induced ICH [26], indicating a beneficial role of endothelial laminin-α5. Consistent with these findings, here we showed that mural cell-derived laminin also contributes to ICH pathogenesis. Given that ablation of astrocytic laminin leads to spontaneous ICH [25], while loss of endothelial laminin-α5 [26] or mural cell-derived laminin only aggravated brain injury in collagenase-induced ICH model, we conclude that astrocytic laminin exerts a more important role in ICH pathogenesis, compared to endothelium- or mural cell-derived laminins.

One important pathological change induced by ICH is microglial activation [67, 74, 75]. We observed increased microglia in PKO mice at the recovery phase after ICH, suggesting that mural cell-derived laminin negatively regulates microglial number. Similarly, mice with laminin-α5 deficiency in endothelial cells displayed higher microglial density after ICH [26]. In addition, loss of laminin γ3 from the retinal vascular plexus significantly increases microglial density and activation in vitro and in vivo [76]. These results suggest that laminin may negatively regulate microglial density and activation.

Unlike control mice, the PKO mice failed to show enhanced brain water content in the ipsilateral hemisphere after ICH. This is mainly due to the abnormally high brain water content in the contralateral hemisphere in these mutants. We further demonstrated elevated brain water content in PKO mice under homeostatic condition, indicating an important role of mural cell-derived laminin in brain water homeostasis. Accumulating evidence suggests that the permeability of cell membrane to water is controlled by a group of water channel proteins called aquaporins, especially AQP4. For example, AQP4 expression in astrocytic endfeet has been shown to be responsible for the development of the cerebral edema during hemorrhagic stroke [77]. In addition, decreased AQP4 expression leads to increased brain water content in cerebral malaria [78]. Consistent with these results, both AQP4 null mice and glial cell-specific AQP4 conditional knockout mice exhibit slightly higher baseline brain water content under homeostatic condition [79, 80]. Furthermore, AQP4 null mice also displayed elevated brain water content in the ipsilateral hemisphere after ICH [81, 82]. In contrast to these studies, we found unchanged AQP4 expression in PKO brains, suggesting that the enhanced baseline brain water content in PKO mice is AQP4-independent. The question then becomes how exactly abrogation of mural cell-derived laminin leads to increased brain water content? One possibility is that mural cell-derived laminin may regulate brain water homeostasis via other AQPs, such as AQP1. It has been reported that AQP1 expression is also involved in cerebral edema formation in hemorrhagic stroke [77]. Another possibility is that mural cell-derived laminin may regulate osmolality between brain parenchyma and the extracellular matrix, the imbalance of which results in abnormal brain water content. These possibilities will be explored in future studies.

The PKO mice were generated by crossing the laminin-γ1^flox/flox^ mice and the PDGFRβ-Cre line. Since PDGFRβ labels both pericytes and vascular smooth muscle cells (vSMCs) in the brain [83, 84], we are unable to distinguish whether the changes in PKO mice are due to loss of pericyte- or vSMC-derived laminin. Given that (1) vSMC-specific laminin-γ1 conditional knockout (termed SKO) mice have a normal lifespan and are grossly normal [24] and (2) PKO but not SKO mice show age-dependent BBB breakdown [28], we speculate that the PKO phenotype reported in this study is more likely to be caused by loss of pericyte- rather than vSMC-derived laminin. However, we cannot exclude the possibility that vSMC-derived laminin also contributes to ICH pathogenesis.

Mural cells predominantly synthesize α4/α5- and γ1-containing laminins [24, 28]. We have shown that mice lacking laminin-α5 in mural cells (α5-PKO) are grossly normal under homeostatic condition [85], while those lacking laminin-γ1 in mural cells develop age-dependent BBB breakdown [28]. The relatively milder phenotype of α5-PKO mice is consistent with the fact that ablation of laminin-γ1 results in loss of both laminin-α4 and -α5 in mural cells [28]. These findings suggest an important role of mural cell-derived laminin-α4 in BBB maintenance under homeostatic condition. In addition, α5-PKO mutants displayed improved outcomes after ischemic stroke [85], suggesting a detrimental role of mural cell-derived laminin-α5 in ischemic stroke; whereas PKO mice showed worse outcomes after ICH, suggesting a beneficial role of mural cell-derived laminin-γ1 in hemorrhagic stroke. This discrepancy could be explained by different stroke models. Ischemic stroke and hemorrhagic stroke have different pathology in the brain [86–88]. It is possible that the different outcomes are due to distinct brain pathology. An alternative explanation is that mural cell-derived laminin-α4 plays a neuroprotective role, while mural cell-derived laminin-α5 plays a detrimental role, with the former being the dominant isoform in mural cells. We are actively testing these possibilities in our laboratory.

## Conclusions

Our results strongly suggest that (1) loss of mural cell-derived laminin exacerbates BBB damage and ICH outcomes, (2) mural cell-derived laminin negatively regulates transcytosis by downregulating caveolin-1 in endothelial cells after ICH, and (3) mural cell-derived laminin regulates brain water content in an AQP4-independent manner.

## Data Availability

All data generated or analyzed during this study are included in this published article.

## Abbreviations

AQP4: Aquaporin-4
BBB: Blood-brain barrier
BM: Basement membrane
BMECs: Brain microvascular endothelial cells
FJC: Fluoro-Jade C
ICH: Intracerebral hemorrhage
PFA: Paraformaldehyde
PKO: Laminin-γ1^flox/flox^:PDGFRβ-Cre^+^
SKO: Laminin-γ1^flox/flox^:transgelin-Cre^+^
TEM: Transmission electron microscopy
TJP: Tight junction protein
vSMC: Vascular smooth muscle cells
